# Physical prognostic factors predicting outcome following lumbar discectomy surgery: systematic review and narrative synthesis

**DOI:** 10.1186/s12891-018-2240-2

**Published:** 2018-09-11

**Authors:** Alison Rushton, Konstantinos Zoulas, Andrew Powell, J. Bart Staal

**Affiliations:** 1 Centre of Precision Rehabilitation for Spinal Pain [CPR Spine] School of Sport, Exercise and Rehabilitation Sciences, University of Birmingham, Birmingham, B15 2TT UK; 2 Polyclinic of Lisieux, Lillebonne, France; 3 ARC Physiotherapy, Saffron Walden, UK; 4 Radboud Institute for Health Sciences, IQ healthcare, Radboud UMC, Nijmegen, 6500 HB The Netherlands; 5 Research group Musculoskeletal Rehabilitation, HAN University of Applied Sciences, Nijmegen, the Netherlands

**Keywords:** Lumbar discectomy, Microdiscectomy, Back pain, Leg pain, Radiculopathy, Prognostic factors, Prognosis, Systematic review, Narrative analysis

## Abstract

**Background:**

Success rates for lumbar discectomy are estimated as 78–95% patients at 1–2 years post-surgery, supporting its effectiveness. However, ongoing pain and disability is an issue for some patients, and recurrence contributing to reoperation is reported. It is important to identify prognostic factors predicting outcome to inform decision-making for surgery and rehabilitation following surgery. The objective was to determine whether pre-operative physical factors are associated with post-operative outcomes in adult patients [≥16 years old] undergoing lumbar discectomy or microdiscectomy.

**Methods:**

A systematic review was conducted according to a registered protocol [PROSPERO CRD42015024168]. Key electronic databases were searched [PubMed, CINAHL, EMBASE, MEDLINE, PEDro and ZETOC] using pre-defined terms [e.g. radicular pain] to 31/3/2017; with additional searching of journals, reference lists and unpublished literature**.** Prospective cohort studies with ≥1-year follow-up, evaluating candidate physical prognostic factors [e.g. leg pain intensity and straight leg raise test], in adult patients undergoing lumbar discectomy/microdiscectomy were included. Two reviewers independently searched information sources, evaluated studies for inclusion, extracted data, and assessed risk of bias [QUIPS]. GRADE determined the overall quality of evidence.

**Results:**

1189 title and abstracts and 45 full texts were assessed, to include 6 studies; 1 low and 5 high risk of bias. Meta-analysis was not possible [risk of bias, clinical heterogeneity]. A narrative synthesis was performed. There is low level evidence that higher severity of pre-operative leg pain predicts better Core Outcome Measures Index at 12 months and better post-operative leg pain at 2 and 7 years. There is very low level evidence that a lower pre-operative EQ-5D predicts better EQ-5D at 2 years. Low level evidence supports duration of leg pain pre-operatively not being associated with outcome, and very low-quality evidence supports other factors [pre-operative ODI, duration back pain, severity back pain, ipsilateral SLR and forward bend] not being associated with outcome [range of outcome measures used].

**Conclusion:**

An adequately powered low risk of bias prospective observational study is required to further investigate candidate physical prognostic factors owing to existing lo*w*/*v*ery-low level of evidence.

**Electronic supplementary material:**

The online version of this article (10.1186/s12891-018-2240-2) contains supplementary material, which is available to authorized users.

## Background

Low back pain [LBP] is the leading cause of disability internationally according to the latest Global Burden of Disease study [1]. A key intervention for LBP with radiculopathy is lumbar discectomy surgery. The number of discectomies performed in community hospitals in the United States in 2012 was 184,000 and the cost of these procedures has doubled in the past 10 years to exceed 9 billion dollars in 2012 [2]. In the UK, the number of lumbar discectomies performed increased from 7043 in 2001–2002 to 8478 in 2013–2014 [3].

Systematic reviews support that lumbar discectomy is superior to prolonged non-surgical treatment for short-term pain relief and improvement in function for lumbar radiculopathy [4, 5]. In the most recent synthesis across trials [using a range of outcome measures], surgical success rates have been estimated as 46–75% patients at 6–8 weeks, and 78–95% patients at 1–2 years post-surgery [6], supporting it as an effective procedure for many patients presenting with radiculopathy; but illustrating variability in outcome for patients. Clinical data also suggest ongoing disability is an issue for some patients, with 30–70% patients reported to experience residual pain [7]. Recent studies also suggest that recurrent lumbar disc herniation can occur, contributing to reoperation [14% from latest figures in the UK [8]], and can often lead to worse outcomes for patients [9, 10].

It is therefore important to determine prognostic factors predicting patient outcome following lumbar discectomy. Knowledge of prognostic factors would inform selection of patients for surgery and selection of patients for rehabilitation following surgery. Prognosis is a developing field of research [11], and findings can contribute to the clinical decision making and evaluation of new methods of patient management [12]. Although an increasing number of primary studies investigating prognostic factors for patient outcome following lumbar discectomy have been published, there are only 3 systematic reviews to date that have synthesised and reviewed the existing evidence.

The first systematic review by den Boer [13] investigated potential biopsychosocial factors across 11 prospective studies. They found that lower level of education, lower work satisfaction, longer duration of sick leave, higher severity of pre-operative pain, higher level of passive avoidance coping strategies, and higher level of psychological problems were associated with poor outcome for patients following lumbar discectomy. Outcome was defined as pain, disability or work capacity or their combination. However, risk of bias was not assessed for the included studies, and heterogeneity of outcome measures and candidate predictors limited both analyses and confidence in the review’s findings, although a basic rating system of the level of evidence was used. In the second systematic review, Sabnis and Diwan [14] investigated the timing of lumbar discectomy across 21 prospective and retrospective studies, and randomised controlled trials. They found that long duration of pre-operative leg pain was associated with poor outcome for patients. However, patient outcome was not clearly defined and risk of bias was not assessed for the included studies [an unsupported scoring system was used to assess aspects of quality], which limits confidence in the review’s findings, although an early best-evidence rating system was used. In the third systematic review, Schoenfeld and Bono [15] investigated the timing of lumbar discectomy surgery across 11 prospective and retrospective studies. They found that a longer duration of pre-operative symptoms was associated with poor patient outcome and identified 6 months duration of symptoms as the critical point when outcome started to be compromised i.e. symptom duration ≥6 months was associated with poor outcome for patients. A range of outcome measures were employed across studies [Short Form Health Survey [SF36], Oswestry Disability Index [ODI], motor weakness, delayed recovery, Visual Analogue Scale [VAS] pain, Japanese Orthopaedic Association Back Pain Questionnaire [JOABPEQ], psychological disorders, degree of return to activities of daily living, pain/disability score [PDS], failed back surgery syndrome, clinical outcome score, good postoperative outcome score, pain and working capacity]. Along with no assessment of risk of bias for the included studies [an unsupported scoring system was used to assess aspects of quality], the heterogeneity of outcome measures and candidate predictors limited analyses and limits confidence in the findings, although an early best-evidence rating system was used.

There is absence of a PRISMA compliant systematic review of prospective cohort studies with a long-term follow-up to synthesise the data investigating in particular, the physical factors that may be associated with patient outcome following lumbar discectomy which are commonly used as indications for surgery [5]. In addition, although early best-evidence rating systems have been used in previous reviews, none have focused on the key issues for this type of review, for example difference in phases of investigation is very relevant to this field of research to ensure a solid theoretical/conceptual model underpinning studies. Identification of physical prognostic factors, which are utilised for clinician’s decision making [16–18], could help inform clinicians which patients are likely to have a more or less favourable outcome. This would allow clinicians to manage their patient’s expectations prior to surgery and help their patient’s make an informed choice about surgery and alternative management strategies.

### Objective

To determine whether pre-operative physical factors are associated with post-operative outcomes in adult patients [≥16 years old] undergoing lumbar discectomy or microdiscectomy.

## Methods

This review was guided by a pre-defined and registered protocol [CRD42015024168], and followed method guidelines of the Back Review Group of the Cochrane Collaboration [19], Cochrane Handbook [20] and PRISMA-P [21]. This systematic review is reported in line with the PRISMA statement [22].

### Eligibility criteria

#### Types of studies

Prospective observational studies with a minimum of 1 year follow up. No restriction was placed on publication date.

#### Participants

Patients [≥16 years old] undergoing first time lumbar discectomy for lumbar disc herniation for irradiating leg pain without a rapid progressive severe motor deficit, cauda equina syndrome or severe comorbid conditions [e.g. arthritis or metabolic bone disease], and with no previous history of other lumbar spine operations.

#### Interventions

Primary, single-level, standard lumbar open discectomy or microdiscectomy.

#### Physical prognostic factors

Pre-operative physical prognostic factors including low back and/or leg pain intensity, duration of low back and/or leg pain, lumbar spine range of motion, disability, quality of life, clinical signs of motor deficit, sensory deficit, straight leg raise [SLR] test, crossed SLR test, walking distance.

#### Outcomes

Outcomes recommended in the evaluation of treatment of spinal disorders [23] were included; specifically disability, physical function, pain intensity and health related quality of life [24, 25].

Exclusion criteria were applied (Table 1).

**Table 1:** Criteria for inclusion and exclusion of studies

Inclusion criteria	
Population	16 years or older, male and female
Intervention	Lumbar and lumbosacral standard open discectomy or microdiscectomy Single one level Primary operation with no previous history of other lumbar spine surgery
Prognostic factors	Intensity of back/leg pain Pre-operative duration of back/leg pain Healh related quality of life Range of movement Motor deficit Sensory deficit SLR Walking distance Disability
Study design	Prospective cohort studies ≥1 year follow up
Exclusion criteria	
Population	History of previous back operation Extraspinal cause of low back and/or leg pain Trauma, vertebral fractures, arthritis or metabolic bone disease, scoliosis, spondylolysis, spondlylolisthesis, spinal stenosis or any other notable non-intervertebral disc abnormalities, trauma, rapid progressive motor deficit, cauda equina syndrome
Intervention	Any other lumbar surgical management Lumbar discectomy combined with other surgery
Study design	Studies not published in English language

### Information sources

A comprehensive search was performed from inception to 31st March 2017 using key databases:

- CINAHL, EMBASE, MEDLINE, PEDro and ZETOC.
- Hand searches of key journals [Spine, European Spine Journal, The Spine Journal].
- Pubmed
- Screening reference lists by hand in papers that match the eligibility criteria.
- Unpublished research: British National Bibliography for Report Literature, Dissertation Abstracts, Index to Scientific and Technical Proceedings, National Technical Information Service, System for Information on Grey Literature.

### Search

There was no restriction of the searches to specific languages. The search strategy was developed by one author [KZ] in discussion with a specialist librarian. It was performed independently by two authors [KZ/AP]. A methodological filter for the identification of prognostic studies which has the greatest sensitivity in Medline [26] was adapted for this study and used in combination with a variety of MESH terms and text words. The concepts that were searched included lumbar disc population, with leg pain and/or low back pain presenting symptoms, lumbar discectomy intervention, and studies investigating prognosis as the methodological focus. The Medline OvidSP search is presented in Table 2 as an example.

**Table 2:** Example of Medline OvidSP Search Strategy

Searches	Results
1	incidence.sh.
2	follow-up studies.sh.
3	prognos*.tw.
4	predict*.tw.
5	course*.tw.
6	1 or 2 or 3 or 4 or 5
7	Lumbar Vertebrae/ or lumbar.mp.
8	Lumbosacral.mp. [m*p* = title, abstract, original title, name of substance word, subject heading word, keyword heading word, protocol supplementary concept word, rare disease supplementary word, unique identifier]
9	Intervertebral Disc/ or low back.mp.
10	7 or 8 or 9
11	sciatica.mp. or exp. Sciatica/
12	radicular pain.mp. or Radiculopathy/
13	Intervertebral Disc Degeneration/ or Intervertebral Disc Displacement/ or degenerative disc disease.mp.
14	Low back pain.mp. or Low Back Pain/
15	11 or 12 or 13 or 14
16	Discectomy.mp. or exp. Discectomy/
17	Discectomy.mp. [m*p* = title, abstract, original title, name of substance word, subject heading word, keyword heading word, protocol supplementary concept word, rare disease supplementary word, unique identifier]
18	Microsurgery/ or microdiscectomy.mp. or Discectomy, Percutaneous/
19	Laminectomy.mp. or Laminectomy/
20	16 or 17 or 18 or 19
21	6 and 10 and 15 and 20

### Study selection

After removing duplicates, screening of the titles and abstracts according to the eligibility criteria (Table 1) was performed independently by 2 authors [KZ/AP] to reduce the risk of excluding relevant studies [27]. Full text articles were obtained for the studies that satisfied the inclusion criteria or in any case where eligibility could not be ascertained from the title or abstract. Full text articles were independently screened by 2 authors [KZ/AP]. Discrepancies about inclusion of articles were resolved by discussion and the third author [AR] was planned to resolve any disagreement.

### Data collection process

Data were extracted from the studies into standardised forms independently by 2 authors [KZ/AP]. The third author [AR] checked the collected data of the included studies**.** Investigators were contacted by email to request additional information for missing or unclearly reported data in included studies.

### Data items

Data were extracted from each study, including: study population, duration of follow up, prognostic factors, outcome measures and key findings.

### Risk of Bias in individual studies

The Quality In Prognostic Studies [QUIPS] tool was used to assess the risk of bias for each individual study. The QUIPS tool was devised for prognostic factor review questions [28] and has demonstrated acceptable inter-rater reliability [median 83.5%] [29]. It consists of 6 categories-domains of potential biases: study participation, study attrition, prognostic factor measurement, outcome measurement, study confounding, statistical analysis and reporting [29]. Each risk of bias domain was rated independently as ‘low’, ‘moderate’ or ‘high’ according to the responses to prompting items, with all domains weighted equally. Overall classification of risk of bias for individual studies was defined as low risk of bias when all domains were rated as low-moderate risk of bias; and high risk of bias when ≥1 domain was rated as high risk of bias [30]. Risk of bias was rated by two authors [KZ/AP] independently. Discrepancies were resolved by discussion and the third author [AR] was available to resolve any disagreement. Inter-rater agreement was planned to be measured with Cohen’s kappa coefficient [31].

### Planned method of analysis

According to the protocol and dependent on homogeneity between the included studies, a quantitative analysis was planned. In the situation where a meta-analysis was not justified [owing to high risk of bias and clinical heterogeneity], a qualitative best evidence synthesis of the results was conducted. This synthesis was based on the risk of bias assessment of the included studies, prognostic factors and the strength of the association with the outcome. Consistency of results across studies was reported to contribute to the overall evidence for an individual candidate prognostic factor. Reporting of multivariable analyses, including odds ratios and 95% Confidence Intervals for dichotomous outcome measures, and βand 95% Confidence Intervals for continuous quantitative outcome measures, and *p* values were reported where possible. The Grading of Recommendations Assessment, Development and Evaluation [GRADE] method [32] was used to rate the overall quality of evidence for a prognostic factor per outcome [e.g. disability], across studies. The GRADE method criteria have been adapted for prognostic factor research [33]. Huguet et al. [33] modified the GRADE domains including 5 factors that may decrease [phase of investigation, study limitations, inconsistency, indirectness, imprecision and publication bias] and 2 factors that may increase [moderate or large effect size [standardized mean difference 0.5–0.8, or odds ratios 2.5–4.25] [33] and exposure-response gradient] the quality level of evidence. As distinct to GRADE used for assessing intervention studies, study design is not a key feature as longitudinal designs are the only option for prognostic research. Phase of investigation is a distinctive GRADE domain for prognostic research with phase 3 explanatory studies [aiming to understand prognostic pathways] and phase 2 explanatory studies [aiming to confirm independent associations between potential prognostic factor and the outcome measure] providing the highest quality of evidence [33].

### Risk of Bias across studies

Visual assessment of potential publication bias with Funnel plots was planned to be performed if > 10 studies with comparable outcome measures were identified.

## Results

### Study selection

**Fig. 1:**
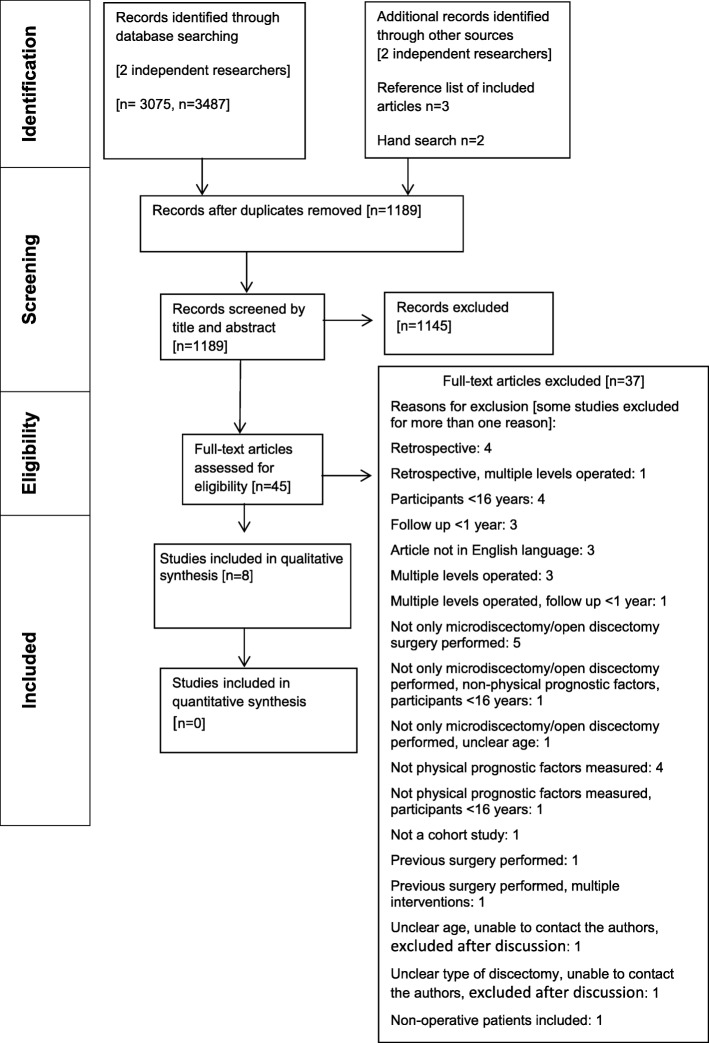
Study selection flow diagram

The initial search resulted in 6567 citations. After exclusion of duplicates, 1189 citations were screened by title and abstract. The full texts of 45 studies were retrieved and assessed for eligibility. Eight studies met the eligibility criteria. Figure 1 shows the number of studies at each stage of selection and the main reasons for exclusion. Details of studies excluded at the full text stage are detailed in the Additional file 1: Table S1. Three non-English studies were excluded at the full text stage. Complete agreement was achieved at each stage of the study selection process following the independent assessments of the 2 authors. Of the 8 included studies, 2 acknowledged that they presented data from the same sample with the later paper by Lewis et al. reporting data at all timepoints [34, 35]. Two further studies appeared to present data from the same sample with the later 2011 article focusing to data on health-related quality of life outcome measures [36, 37]. A request for clarification from the authors did not receive a response. In both cases, data are presented as the same study to ensure appropriate weighting of the evidence in the narrative synthesis. Overall therefore, 6 studies were included reflecting 8 articles.

### Study characteristics

The main characteristics of the 6 included studies are presented in Table 3.

**Table 3:** Study characteristics

Study	Country	Characteristics of Participants	Follow-up	Physical Prognostic factors	Outcome measures
Divecha et al., 2014	United Kingdom	*n* = 89 Age: 25–79, mean 48.6 Gender: Male *n* = 46 [51.7%] Female *n* = 43 [48.3%]	1 year *n* = 32 [35%]	• Pre-operative leg pain [% of pain that was radicular, calculated from the Core Outcome Measures Index [COMI]	• COMI score – patient completed assessment through Spine Tango. Includes questions on the severity of leg and back pain. • Definition of outcome unclear for multivariate analyses
Fisher et al., 2004	Canada	*n* = 82 Age: 17–83, mean 42.2 Gender: Male *n* = 52 [63.4%] Female *n* = 30 [36.6%]	1 year *n* = 71 [87%]	• Pre-operative duration of leg pain in months	• Health Related Quality Of Life [HRQOL] outcome comprising: a. North American Spine Society instruments: Neurogenic Symptom Score and Pain/Disability Score b. Short Form-36 [SF-36] questionnaire
Lewis et al., 1987 (and Weir, 1979)	Canada	*n* = 100 Age^a^: Mean [SD] 41.7 [1] Gender: Male 75% Female 25%	1 year *N* = 91 [91%] 5–10 years *n* = 81 [81%]	• Pre-operative duration of leg pain in months • Ipsilateral straight leg raise (detail of measurement not reported) • Forward bend (detail of measurement tool not reported)	• Relief of back pain • Relief of leg pain • No multivariate analyses
Nygaard et al., 2000	Norway	n = 132 Age^a^: > 18 Gender: Not reported	1 year *n* = 132 [100%]	• Pre-operative duration of leg pain in months • Pre-operative duration of back pain in months	• Clinical Overall Score, calculated from 40% weighting pain, 20% clinical examination, 20% functional status [Oswestry Disability Index, ODI] and 20% analgesia
Silverplats et al., 2010	Sweden	*n* = 171 Age^a^: Mean[SD] 39 [11] Gender: Male *n* = 95 [55.6%] Female *n* = 76 [44.4%]	2 years *n* = 154 [90%] Mean[SD] long term 7.3 [1.0] years Range 5.1–9.3 years *n* = 140 [81%]	• Pre-operative leg pain - recorded with three 0–100 Visual Analogue Scale [VAS] representing ‘pain when as worst’, ‘pain when as least’ and ‘pain right now’. Mean value of the three scales recorded • Pre-operative back pain - recorded with three 0–100 VAS representing ‘pain when as worst’, ‘pain when as least’ and ‘pain right now’. Mean value of the three scales recorded • Pre-operative duration of leg pain in months • Pre-operative ODI- self complete questionnaire 0–100	Primary outcomes: • MacNab classification of post-operative outcome [at 2 years] with 4 categories of outcome – excellent, good, fair, poor but unclear how applied as dichotomized outcome in multivariate analyses • Satisfaction with treatment [satisfied, partly, not satisfied, both follow up points] Secondary outcomes: • Change in leg pain [improved, no improvement, worse] • Change in back pain [improved, no improvement, worse]
Silverplats et al., 2011	Sweden	*n* = 117 Age: 18–66, mean 39 Gender: Male *n* = 63 [54%] Female *n* = 54 [46%]	Range 5–8 years 2 years 82% 7 years 76%	• Pre-operative duration of leg pain in months • Pre-operative leg pain [detail of measurement not reported] • Pre-operative back pain [detail of measurement not reported] • Pre-operative EuroQol-5 Dimension [EQ-5D] score for HRQOL self-completion questionnaire 0–100	• Change in EQ- 5D score
Solberg et al., 2005	Norway	*n* = 228 Age: Mean[SD] 41 [11] Gender: Male *n* = 114 [63.3%] Female *n* = 66 [36.7%]	1 year *n* = 180 [78.9%]	• Pre-operative ODI score - self complete questionnaire 0–100 • Pre-operative duration of leg pain in months • Pre-operative duration of back pain in months • Pre-operative leg pain 0–100 VAS no pain to worst conceivable pain • Pre-operative back pain 0–100 VAS no pain to worst conceivable pain	Primary outcome: • ODI score classified as: a. deterioration [increased ODI] or no deterioration [decreased/unchanged ODI] b. poor [ODI > 39] or good [ODI < 40] Secondary outcomes: • VAS back pain • VAS leg pain

^a^ After communication with the authors it was confirmed that all participants were ≥ 16 years old

NOTE: Silverplats et al. 2010 and 2011 reported as two separate rows for clarity of prognostic factors and outcomes

#### Methods

The studies were conducted in four different countries and published between 1979 and 2011. Five studies were published, 1 was unpubished [38] but was presented at a conference and data were acquired after personal communication with the authors. The follow-up period in included studies ranged from 1 to 10 years.

#### Participants

The total number of participants included across the 6 studies was *n* = 802 and sample sizes ranged from 82 to 228. Age ranged from 17 to 83 years. After communication with the authors of 3 studies that did not report the age range of the participants [34, 36, 39], it was confirmed that all participants were ≥ 16 years old to enable study inclusion.

#### Physical prognostic factors

The most common physical prognostic factor that was investigated in 5 studies [34–37, 39–41] was pre-operative duration of leg pain, followed by intensity of pre-operative leg pain investigated in 3 studies [36–38, 41], and pre-operative back pain investigated in 2 studies [36, 37, 41].

#### Outcome measures

The range of outcome measures included: VAS for pain, ODI, EuroQol-5 Dimension [EQ-5D] score, SF-36, Neurogenic Symptom Score [NSS] and PDS for quality of life, Core Outcome Measures Index [COMI], Clinical Overall Score [COS], MacNab classification of postoperative outcome, satisfaction with treatment and change in leg/back pain.

### Risk of Bias within studies

Of the 6 included studies, 1 was assessed as low risk of bias and 5 as high risk of bias (Table 4). Complete agreement in the assessment of risk of bias in all domains was achieved between the 2 authors. The domain ‘study attrition’ was rated as high risk of bias in 5 of the studies and only the domain ‘outcome’ was rated as low risk of bias in all studies. Most studies did not account for all of the important potential confounders in their study design and the risk of selection bias was also high due to incomplete reporting.

**Table 4:** Methodological Assessment according to six domains of potential biases [QUIPS]^27^

Study [*n* = 6]	Study Participation	Study Attrition [Follow-up]	Prognostic Factor	Outcome	Confounding Factor	Analysis	Overall Risk of Bias
Divecha et al., 2014	High	High	Moderate	Low	Moderate	Low	High
Fischer et al., 2004	Moderate	High	Moderate	Low	Low	Moderate	High
Lewis et al., 1987 and Weir 1979	High	High	Moderate	Low	High	High	High
Nygaard et al., 2000	High	High	Moderate	Low	High	High	High
Silverplats et al., 2010 and Silverplats et al., 2011	Moderate	Moderate	Low	Low	Low	Low	Low
Solberg et al., 2005	Moderate	High	Low	Low	High	Moderate	High

A study was considered to be of low risk of bias when all domains were rated as low-moderate risk of bias

A study was considered to be of high risk of bias when ≥1 domain[s] were rated as high risk of bias

### Results per physical prognostic factor

Eight different physical prognostic factors were investigated (Table 5). Due to heterogeneity between the included studies [predictors, follow-up timepoints, outcome measures] a meta-analysis was not justified, and a qualitative best evidence synthesis of the results was performed. In particular, there was great diversity in the patient outcomes assessed in the included studies. Using the adapted GRADE method for prognostic research [33] to rate the overall quality of evidence, all included studies were phase 1 predictive modelling or explanatory studies carried out to generate a hypothesis, and consequently the quality of evidence was moderate as a starting point (Table 6) [33]. The level of evidence was downgraded in particular for inconsistency, and only upgraded for effect size for 2 prognostic factors.

**Table 5:** Overview of Significant Physical Prognostic Factors: synthesis across included studies [bivariate and multivariable analyses when reported are documented here for consistency - reporting was inconsistent across studies]

Physical prognostic factors	Study and risk of bias	Results	Summary of study findings [based on multivariate analyses; where significant, direction of effect is reported]	gy9	Summary of findings across studies
Oswestry Disability Index [ODI]	Silverplats et al., 2010 LOW risk of bias	Bivariate analyses: ● Patients with worse pre-operative ODI scores were more likely to report improvement in leg pain [dichotomized as improvement versus no improvement / worse]. Patients with improved leg pain had pre-operative mean ODI 52 compared to 42 in no improvement/worse group [*p* = 0.040]. ● Patients with worse pre-operative ODI scores were more likely to report improvement in back pain [dichotomized as improvement versus no improvement / worse]. Patients with improved back pain had pre-operative mean of 52 compared to 44 in no improvement/worse group [*p* = 0.040]. Multivariable analyses: ● ODI was not a significant predictor when using the full model of potential predictors [no measure of association reported] at 2 years or long term follow up.	Pre-operative ODI was not significant as a prognostic factor for leg pain or for back pain at 2 years or long term follow up [mean 7.3 ± 1.0 years].	+ Very low	Using GRADE, there is very low level evidence that ODI is not associated with patient outcome.
	Solberg et al., 2005 HIGH risk of bias	Multivariable analyses: ● Using change in ODI score as a dichotomous variable (deterioration or no deterioration of score) in binary stepwise logistic regression analyses, a low pre-operative ODI score was an independent risk factor for ‘deterioration’ [β [age adjusted] 0.087, *p* = 0.011; β [independent risk factor] –0.216, *p* = 0.013]. ● Using ODI raw score at 12 months as a dichotomous variable [“good” ODI score > 39, or “poor” outcome] pre-operative ODI was not an independent risk factor for a “poor” outcome [no measure of association reported]. Lower ODI score [β = − 0.0442, *p* < 0.001] pre-operatively was a predictor of less improvement in ODI score.	Pre-operative ODI was significant as a prognostic factor for post-operative disability [ODI] at 12 months. *Higher pre-operative ODI predicts better outcome [lower ODI] at 12 months.*		
Duration of back pain	Nygaard et al., 2000 HIGH risk of bias	● Multivariable analyses: Multiple linear regression analysis demonstrated that pre-operative duration of back pain was not predictive of clinical overall score [COS]; coefficient β [Standard error] = − 0.26 [0.16], t test − 1.65, *p* = 0.100.	Pre-operative duration of back pain was not significant as a prognostic factor for COS at 12 months.	+ Very low	Using GRADE, there is very low level evidence that duration of back pain is not associated with patient outcome.
	Solberg et al., 2005 HIGH risk of bias	Multivariable analyses: ● Using change in ODI score as a dichotomous outcome variable in binary stepwise logistic regression analyses, duration of back pain was not an independent risk factor for ‘deterioration’ [β [age adjusted] 0.001, *p* = 0.304]. ● Using ODI raw score at 12 months as a dichotomous variable [“good” ODI score > 39, or “poor” outcome] in multivariate analyses, duration of back pain was not an independent risk factor for a “poor” outcome [no measure of association reported].	Pre-operative duration of back pain was not significant as a prognostic factor for disability [ODI] at 12 months.		
Duration of leg pain	Fischer et al., 2004 HIGH risk of bias	Multivariable analyses: Patients with longer pre-operative duration of leg pain were more likely to report less improvement in Pain Disability Score [PDS] [*p* = 0.026] after adjustment for gender, age and pre-operative PDS. Mean change PDS 24.4 for duration 0–3 months, 20.0 for duration 3.1–9 months, 13.1 for duration > 9 months [no measures of association reported].	Pre-operative duration of leg pain was significant as a prognostic factor for PDS at 12 months. *Shorter pre-operative duration of leg pain predicts better outcome [lower PDS] at 12 months.*	a	am
	Lewis et al., 1987 and Weir et al., 1979 HIGH risk of bias	Bivariate analyses: ● Duration leg pain < 17 months associated with complete relief of back pain in 43/71 cases [61%] at 1 year; 39/65 cases [60%] at 5–10 years. ● Duration leg pain ≥17 months associated with complete relief of back pain in 12/19 cases [63%] at 1 year; 9/15 cases [60%] at 5–10 years. ● Duration leg pain < 17 months associated with complete relief of leg pain in 54/71 cases [76%] at 1 year; 43/65 cases [66%] at 5–10 years. ● Duration leg pain ≥17 months associated with complete relief of leg pain in 12/19 cases [63%] at 1 year; 6/15 cases [40%] at 5–10 years. ● Significant association [chi-square of Fisher’s exact test] at 1-year follow-up review between duration leg pain and relief of back or leg pain [above]. Shorter duration of leg pain before surgery is associated with relief of leg pain following surgery. Not significant at 5–10 years [results not reported].	Pre-operative duration of leg pain was not significant as a prognostic factor for leg pain and for back pain at 12 months [no multivariable analyses].		
	Nygaard et al., 2000 HIGH risk of bias	Multivariable analyses: ● Patients with longer pre-operative duration of leg pain were more likely to report less improvement in COS. Multiple linear regression analysis, coefficient β [Standard error] = 0.98 [0.3], t test 3.23, *p* = 0.0016.	Pre-operative duration of leg pain was significant as a prognostic factor for COS at 12 months. *Shorter pre-operative duration of leg pain predicts better outcome [lower COS] at 12 months.*		
	Silverplats et al., 2010 LOW risk of bias	Bivariate analyses: ● Patients with longer pre-operative duration of leg pain were more likely to report improvement in leg pain. Pre-operative short duration [< 6 months] of leg pain predicts good outcome on MacNab [dichotomized outcome] classification [*p* = 0.039] at 2-year follow up and predicts patient satisfaction with treatment [*p* = 0.019] at long term follow-up [mean 7.3 ± 1.0 years]. Multivariable analyses: ● Duration of leg pain was not a significant predictor when using the full model of potential predictors [no measure of association reported].	Pre-operative duration of leg pain was not significant as a prognostic factor for leg pain or health-related quality of life [EQ-5D] at 2 year and long term follow up [mean 7.3 ± 1.0 years].		
	Silverplats et al., 2011 LOW risk of bias	Multivariable analyses: ● Duration of leg pain was not a significant predictor for EuroQol-5 Dimension, EQ-5D at 2 years [no measure of association reported].			
	Solberg et al., 2005 HIGH risk of bias	Multivariable analyses: ● Duration of leg pain was not an independent risk factor for ‘deterioration’ [β [age adjusted] 0.008, *p* = 0.006; β [independent risk factor] 0.005, *p* = 0.572]; using change in ODI score as a dichotomous outcome variable (deterioration or no deterioration). ● Using ODI raw score at 12 months as a dichotomous outcome variable [“good” ODI score > 39, or “poor” outcome] duration of leg pain was not an independent risk factor for a “poor” outcome [no measure of association reported].	Pre-operative duration of leg pain was not significant as a prognostic factor for disability [ODI] at 12 months.		
Severity leg pain	Divecha et al., 2014 HIGH risk of bias	Bivariate analyses: ● Patients with worse pre-operative leg pain were more likely to report improvement in functional outcome. Pearson’s correlation coefficient between pre-operative leg pain [%] and Core Outcome Measures Index [COMI] score at 12 months was −0.394 (95% CI -0.653, − 0.053; *p* = 0.0256]. Multivariable analyses: ● Patients with higher pre-operative leg pain had significantly lower COMI [R^2^ = 0.155, *p* = 0.03] at 12 months.	Pre-operative severity of leg pain was significant as a prognostic factor for functional outcome [COMI] at 12 months. *Higher severity pre-operative leg pain predicts better outcome [lower COMI] at 12 months.*	++ Low	Using GRADE, there is low level evidence that higher severity of pre-operative leg pain predicts better Core Outcome Measures Index at 12 months and better post-operative leg pain at 2 and 7 years.
	Silverplats et al., 2010 LOW risk of bias	Bivariate analyses: ● Patients with higher pre-operative leg pain were more likely to report improvement in leg pain. Patients with improved leg pain had higher leg pain pre-operatively on VAS [60 versus 47, *p* = 0.008] Multivariable analyses: ● For improvement in leg pain the only significant predictor among all potential predictors was pre-operative VAS leg pain (*p* = 0.039). Pre-operative VAS leg pain was also the first and only predictor selected by the stepwise procedure [no measure of association reported].	Pre-operative severity of leg pain was significant as a prognostic factor for leg pain at 2 years and long term follow up [mean 7.3 ± 1.0 years]. Pre-operative severity of leg pain was not significant as a prognostic factor for EQ-5D at 2 years or long term follow up [mean 7.3 ± 1.0 years]. *Higher severity pre-operative leg pain predicts better outcome [lower leg pain] at 2 years and long term follow up [mean 7.3 ± 1.0 years].*		
	Silverplats et al., 2011 LOW risk of bias	Bivariate analyses: Patients with higher pre-operative leg pain were more likely to report improvement in health-related quality of life. Pre-operative VAS leg pain was correlated with change in EQ-5D at 2-year follow-up [*r* = 0.33, *p* = 0.002] and at 7-year follow up [*r* = 0.23, *p* = 0.04]. Multivariable analyses: VAS leg pain was not identified as a significant predictor of EQ-5D [no measure of association reported].			
	Solberg et al., 2005 HIGH risk of bias	Bivariate analyses: Patients with higher pre-operative leg pain were more likely to report improvement in disability. Pre-operative VAS leg pain mean [SD; 95%CI] was 63.4 [27.5; 59.3 to 67.4], and at 12 months was 16.8 [21.1; 13.7 to 20.0]. Improvement was 46.5 [33.4, 41.6 to 51.4]. Multivariable analyses: Using change in ODI score as a dichotomous outcome variable, VAS leg pain was not an independent risk factor for ‘deterioration’ [β [age adjusted] -0.009, *p* = 0.481] at 12 months. Using ODI raw score at 12 months as a dichotomous outcome variable [“good” ODI score > 39, or “poor” outcome] VAS leg pain was not an independent risk factor for a “poor” outcome [no measure of association reported].	Pre-operative severity of leg pain was not significant as a prognostic factor for disability [ODI] at 12 months.		
Severity back pain	Silverplats et al., 2010 LOW risk of bias	Bivariate analyses: Patients with higher pre-operative back pain were more likely to report improvement in back pain. Patients with improved back pain had higher VAS back pain pre-operatively [53 versus 36, *p* = 0.001]. Multivariable analyses: Pre-operative back pain was not a significant predictor when [no measure of association reported] at 2 years or long term follow up [mean 7.3 ± 1.0 years].	Pre-operative severity of back pain was not significant as a prognostic factor for back pain or EQ-5D at 2 years or long term follow up [mean 7.3 ± 1.0 years].	+ Very low	Using GRADE, there is very low level evidence that severity of back pain is not associated with patient outcome.
	Silverplats et al., 2011 LOW risk of bias	Bivariate analyses: Back pain at baseline was not significantly correlated with change in EQ-5D at any follow-up. Multivariable analyses: Back pain was not identified as a significant predictor of EQ-5D at 2 years follow up [no measure of association reported].			
	Solberg et al., 2005 HIGH risk of bias	Bivariate analyses: Baseline VAS back pain (0–100 points) mean [SD; 95%CI] = 51.7 [29.3; 47.4, 56.0]. 12 months 21.3 [22.6; 18.0, 24.6]. Improvement 31.4 [35.6, 25.2–35.6]. VAS back pain pre-operatively not predictive of follow up ODI score at 12 months. Multivariable analyses: VAS back pain was not an independent risk factor for ‘deterioration’ [β–[age adjusted] 0.003, *p* = 0.800]; using change in ODI score as a dichotomous variable [deterioration or no deterioration]. Using ODI raw score at 12 months as a dichotomous variable [“good” ODI score > 39, or “poor” outcome], VAS back pain was not an independent risk factor for a “poor” outcome [no measure of association reported].	Pre-operative severity of back pain was not significant as a prognostic factor for disability [ODI] at 12 months.		
Health-related quality of life [EuroQol-5 Dimension, EQ-5D]	Silverplats et al., 2011 LOW risk of bias	Bivariate analyses: Patients with lower pre-operative EQ-5D were more likely to report improvement in health-related quality of life [EQ-5D]. Pre-operative EQ-5D was correlated with change in EQ-5D at 2-year or 7-year follow-ups [*r* = −0.70, *p* < 0.001 and *r* = − 0.71, *p* < 0.001]. Multivariable analyses: The only significant predictor of outcome was pre-operative EQ-5D score. The influence of baseline EQ-5D score was estimated [β = − 1.0, 95% CI: − 1.2, − 0.8] at 2 years.	Pre-operative EQ-5D was significant as a prognostic factor for health-related quality of life [EQ-5D] at 2 years. *Lower pre-operative EQ-5D predicts better outcome [lower EQ-5D] at 2 years.*	+ Very low	Using GRADE, there is very low level evidence that a lower pre-operative EQ-5D predicts better EQ-5D at 2 years.
Ipsilateral Straight Leg Raise [SLR]	Lewis et al., 1987 and Weir, 1979 HIGH risk of bias	Bivariate analyses: Positive ipsilateral SLR associated with complete relief of back pain in 47/75 cases [63%] at 1 year; 41/69 cases [59%] at 5–10 years. Negative ipsilateral SLR associated with complete relief of back pain in 9/16 cases [56%] at 1 year; 8/12 cases [67%] at 5–10 years. Positive ipsilateral SLR associated with complete relief of leg pain in 59/75 cases [79%] at 1 year; 42/69 cases [61%] at 5–10 years. Negative ipsilateral SLR associated with complete relief of leg pain in 8/16 cases [50%] at 1 year; 8/12 cases [67%] at 5–10 years. Significant association [chi-square of Fisher’s exact test] at 1-year follow-up review between ipsilateral SLR and relief of back or leg pain [above]. Positive ipsilateral SLR before surgery is associated with relief of back and leg pain following surgery. Not significant at 5–10 years [results not reported].	Pre-operative ipsilateral SLR was not significant as a prognostic factor for back pain or leg pain at 5–10 years [no multivariable analyses].	+ Very low	Using GRADE, there is very low level evidence that straight leg raise is not associated with patient outcome.
Forward bend	Lewis et al., 1987 and Weir, 1979 HIGH risk of bias	Bivariate analyses: Forward bend to knee associated with complete relief of back pain in 41/58 cases [71%] at 1 year; 33/50 cases [66%] at 5–10 years. Forward bend to mid tibia or floor associated with complete relief of back pain in 15/33 cases [45%] at 1 year; 16/31 cases [52%] at 5–10 years. Forward bend to knee associated with complete relief of leg pain in 48/58 cases [83%] at 1 year; 34/50 cases [68%] at 5–10 years. Forward bend to mid tibia or floor associated with complete relief of leg pain in 19/33 cases [58%] at 1 year; 13/31 cases [42%] at 5–10 years. Significant association [chi-square of Fisher’s exact test] at 1-year follow-up review between forward bend and relief of back or leg pain [above]. Positive forward bend to knee before surgery is associated with relief of back and leg pain following surgery. Not significant at 5–10 years [results not reported].	Pre-operative forward flexion was not significant as a prognostic factor for back pain or leg pain at 5–10 years [no multivariable analyses].	+ Very low	Using GRADE, there is very low level evidence that forward bend is not associated with patient outcome.

NOTE: Silverplats et al. 2010 and 2011 reported as two separate rows for clarity of prognostic factors and outcomes but combined from ‘summary on study findings’ column onwards when both studies have reported on a single prognostic factor

**Table 6:** Adapted Grading^31^ of Recommendations Assessment, Development and Evaluation [GRADE] table for systematic reviews with meta-analysis of prognostic studies for positive outcome across a range of measures

GRADE factor													
						Factors that may reduce the quality					Factors that may increase the quality		
Prognostic factor [pre-operative measures]	Number of participants	Number of studies	Number of cohorts	Estimated effect size [95% CI]	Phase (design)	Study limitations	Inconsistency	Indirectness	Imprecision	Publication bias	Moderate/ large effect size	Dose effect	Overall quality
Oswestry Disability Index [ODI]	399	2	2	Unclear	1	✓	x	✓	x	x	x	x	+ Very low
Duration of back pain	360	2	2	Unclear	1	x	✓	✓	x	x	✓	x	+ Very low
Duration of leg pain	713	5	5	Unclear	1	✓	x	✓	✓	✓	x	x	++ Low
Severity leg pain	488	3	3	Unclear	1	✓	x	✓	✓	✓	x	x	++ Low
Severity back pain	399	2	2	Unclear	1	✓	x	✓	x	x	✓	x	+ Very low
EuroQol-5 Dimension [EQ-5D]	140	1	1	Unclear	1	✓	x	✓	✓	x	x	x	+ Very low
Ipsilateral Straight Leg Raise [SLR]	100	1	1	Unclear	1	x	x	✓	x	x	x	x	+ Very low
Forward bend	100	1	1	Unclear	1	x	x	✓	x	X	x	x	+ Very low

GRADE factors: ✓, no serious limitations; X, serious limitations [or not present for moderate/large effect size]; unclear, unable to rate item based on available information. For overall quality of evidence: +, very low; ++, low; +++, moderate; ++++, high

### ODI

The ODI was included in 2 studies [36, 41] as a candidate prognostic factor. There were inconsistencies regarding the association between the ODI and several outcomes. One study [low risk of bias] found no association [leg pain or back pain at 2 or 7 years], while 1 study [high risk of bias] found that higher disability was associated with better patient outcome [ODI at 12 months]. Using GRADE, there is very low level evidence that ODI is not associated with patient outcome.

### Duration back pain

Duration of back pain was included in 2 studies [39, 41] as a candidate prognostic factor. Consistent findings from the 2 studies [high risk of bias] found no association with patient outcome [Clinical Overall Score and ODI at 12 months]. Using GRADE, there is very low level evidence that duration of back pain is not associated with patient outcome.

### Duration leg pain

Pre-operative duration of leg pain was included in 5 studies [34–39, 41] as a candidate prognostic factor. There were inconsistencies regarding the association between the duration of pre-operative leg pain and numerous outcomes. Three studies [1 low risk of bias and 2 high risk of bias] found no association [leg pain, back pain and ODI at 12 months; leg pain and health-related quality of life at 2 and 7 years] while 2 studies [both high risk of bias] found that shorter pain duration was associated with better patient outcome [Pain Disability Score and Clinical Overall Score at 12 months]. Using GRADE, there is low level evidence that duration of pre-operative leg pain is not associated with patient outcome.

### Severity leg pain

Severity of leg pain was included in 3 studies [36–38, 41] as a candidate prognostic factor. There were inconsistencies regarding the association between the severity of leg pain and several outcomes. Two studies [1 low risk of bias and 1 high risk of bias] found no association [health related quality of life at 2 and 7 years; ODI at 12 months], while 2 studies [1 low risk of bias and 1 high risk of bias] found that higher severity of leg pain was associated with better patient outcome [leg pain at 2 and 7 years; Core Outcome Measures Index at 12 months]. Using GRADE, there is low level evidence that higher severity of pre-operative leg pain predicts better Core Outcome Measures Index at 12 months and better post-operative leg pain at 2 and 7 years.

### Severity back pain

Severity of back pain was included in 2 studies [36, 37, 41] as a candidate prognostic factor. Consistent findings from the 2 studies [1 low risk of bias and 1 high risk of bias] found no association with patient outcome [ODI at 12 months; back pain and health-related quality of life at 2 and 7 years]. Using GRADE, there is very low level evidence that severity of back pain is not associated with patient outcome.

### Health-related quality of life

Health-related quality of life [EQ5D] was included in 1 study [37] as a candidate prognostic factor. The study [low risk of bias] found that low quality of life pre-operatively was associated with better patient outcome [health-related quality of life at 2 years]. Using GRADE, there is very low level evidence that a lower pre-operative EQ-5D predicts better EQ-5D at 2 years.

### Straight leg raise and forward bend

Ipsilateral straight leg raise and forward bend were included in 1 study [34] as candidate prognostic factors. The study [high risk of bias] found that ipsilateral straight leg raise and forward bend were not associated with patient outcome [back pain or leg pain at 5–10 years]. Using GRADE, there is very low level evidence that straight leg raise and forward bend are not associated with patient outcome.

## Discussion

This is the first systematic review of physical prognostic factors to evaluate their association with patient outcome following lumbar discectomy. Only 6 studies were included, and risk of bias in the included studies was disappointing with only 1 study at low risk of bias. As a consequence, our current understanding of physical prognostic factors is limited.

Based on the strength of association of the prognostic factors investigated and the overall quality of evidence, we know that pre-operative severity of leg pain [low level of evidence] and quality of life [very low level of evidence] are associated with patient outcome. Specifically, higher severity pre-operative leg pain predicts better Core Outcome Measures Index at 12 months and better leg pain at 2 and 7 years; and lower pre-operative EQ-5D predicts better EQ-5D at 2 years. The findings are consistent with den Boer’s previous review that found higher severity of pre-operative pain was associated with patient outcome [13]. Greater confidence in low risk of bias studies in situations of inconsistency between study findings contributed to severity of leg pain being identified overall as associated with patient outcome and this may be a limitation of this review. Interestingly, apart from the Core Outcome Measures Index, for both significant factors the prognostic factor and outcome were the same measure, and therefore for both of these factors, the reason they were more likely to report improvement could be due to the fact that were starting from a higher level of pain or lower level of quality of life initially.

Other potential predictors examined were pre-operative ODI, duration leg pain, duration back pain, severity back pain, ipsilateral SLR and forward bend, and very low quality of evidence found that they were not associated with patient outcome, except for duration of leg pain where the quality of evidence was low. Consistent findings identified that pre-operative duration of back pain and severity of back pain were not associated with patient outcome [clinical overall score and ODI at 12 months; back pain or EQ-5D at 2 or 7 years and ODI at 12 months respectively]. Findings from 1 study [34, 35] identified that pre-operative ipsilateral SLR and forward bend were not associated with patient outcome, although it is difficult to have any confidence in these findings as they were based on bivariate analyses only [Table 5]. Inconsistent findings identified that pre-operative ODI [1 low risk of bias, 1 high risk of bias study] was not associated with patient outcome [leg pain or back pain at 2 and 7 years; ODI at 12 months]. None of these factors had been examined in previous reviews. Inconsistent findings identified that duration leg pain was not associated with patient outcome [Pain Disability Score, ODI, leg pain, back pain and Clinical Overall Score at 12 months; EQ-5D at 2 and 7 years]. This was in contrast to previous reviews that identified pre-operative duration of leg pain as associated with patient outcome [14, 15]. It is however difficult to have confidence in the findings from previous reviews as they themselves were at risk of bias.

In comparison with other systematic reviews [13–15], this review included only prospective cohort studies which are the gold standard design for investigating prognostic factors to enable optimal measurement of outcomes and predictors [42]. Our findings illustrate that the current level of evidence is lo*w*/*v*ery low. An adequately powered low risk of bias prospective observational study that assesses patient outcome at 12 months following surgery is required to further investigate pre-operative severity of leg pain, EQ-5D and duration of leg pain; and those candidate prognostic factors with inconsistent and very low level evidence to date, specifically ODI, duration back pain, severity back pain, ipsilateral SLR and forward bend. Other physical factors worthy of investigation and examined in studies excluded from this review, include pre-operative motor deficit, sensory loss and walking capacity.

### Strengths and limitations

This is the first low risk of bias systematic review [self-assessed using AMSTAR 2 [43]] that has synthesised the evidence for physical prognostic factors predicting patient outcome following lumbar discectomy surgery. However, the review is limited by risk of bias across the small number of available studies, and a lack of comparable outcome measures across studies. This lack of comparable outcome measures meant that the definition of outcome taken into the GRADE analysis was broad encompassing a range of domains and outcome measures. The exclusion of 3 non-English studies could be a major limitation of this review as key findings may have been missed; particular as only 6 studies were included. Discussion of this review’s findings is limited by the scarce literature in this area and the quality of reporting of individual study results which was inconsistent and poor overall.

## Conclusions

Results from this systematic review identified low level evidence that higher severity of pre-operative leg pain predicts better Core Outcome Measures Index at 12 months and better post-operative leg pain at 2 and 7 years. There is very low level evidence that a lower pre-operative EQ-5D predicts better EQ-5D at 2 years. Low level evidence supports duration of leg pain pre-operatively not being associated with outcome, and very low-quality evidence supports other factors [pre-operative ODI, duration back pain, severity back pain, ipsilateral SLR and forward bend] not being associated with outcome [range of outcome measures used]. Research to date is however poor, consisting mostly of high risk of bias studies with inadequate reporting of analyses, not enabling full understanding of the prognostic value of physical factors assessed prior to surgery.

An adequately powered low risk of bias prospective observational study, with clear reporting of multivariable analyses is required to investigate all potential physical factors. Knowledge of the physical prognostic factors is essential to inform clinical decision-making processes regarding selection of patients for surgery and potentially the targeting of patients for rehabilitation following surgery. The results of prospective observational studies can help clinicians to decide which people should receive surgery or rehabilitation. However, a limitation is that a difference in prognosis does not necessarily mean a causal link with the surgery. Therefore, when we understand the prognostic factors we need to investigate them in a randomised controlled trial to investigate predictors of treatment response.

## Additional file

Additional file 1: **Table S1.** Studies excluded at full text review stage with reasons (*n* = 37). (DOCX 45 kb)

## Abbreviations

COMI: Core Outcome Measures Index
COS: Clinical Overall Score
EQ5D: EuroQol-5 Dimension
JOABPEQ: Japanese Orthopaedic Association Back Pain Questionnaire
LBP: Low back pain
NSS: Neurogenic Symptom Score
ODI: Oswestry Disability Index
PDS: Pain disability score
QUIPs: Quality In Prognostic Studies
SF36: Short Form Health Survey
SLR: Straight leg raise
WAS: Visual Analogue Scale
