# Correction to: Physical prognostic factors predicting outcome following lumbar discectomy surgery: systematic review and narrative synthesis

**DOI:** 10.1186/s12891-018-2288-z

**Published:** 2018-10-15

**Authors:** Alison Rushton, Konstantinos Zoulas, Andrew Powell, J B Staal

**Affiliations:** 1 Centre of Precision Rehabilitation for Spinal Pain [CPR Spine] School of Sport, Exercise and Rehabilitation Sciences, University of Birmingham, Birmingham, B15 2TT UK; 2 Polyclinic of Lisieux, Lillebonne, France; 3 ARC Physiotherapy, Saffron Walden, UK; 4 Radboud Institute for Health Sciences, IQ healthcare, Radboud UMC, Nijmegen, 6500 HB The Netherlands; 5 Research group Musculoskeletal Rehabilitation, HAN University of Applied Sciences, Nijmegen, the Netherlands

## Correction

Following publication of the original article [1], the author reported that the tagging of their name had been done incorrectly during production process. The original article has been corrected.

**Incorrect tagging:**

**Bart Staal J**

**Correct tagging:**

**Staal JB**
