# Supplementary material for: Physical prognostic factors predicting outcome following lumbar discectomy surgery: systematic review and narrative synthesis
Source: BMC Musculoskelet Disord. 2018 Sep 11;19:326. doi: 10.1186/s12891-018-2240-2 (PMC6134506; doi:10.1186/s12891-018-2240-2)
Supplement: Supplementary file 1 — Table S1. Studies excluded at full text review stage with reasons (n = 37). (DOCX 45 kb) [file 12891_2018_2240_MOESM1_ESM.docx]

**Table S1 Supplementary information: Studies excluded at full text review stage with reasons (n=37)**

| **Authors** | **Title** | **Journal** | **Year** | **Vol** | **Page** | **Reason(s) for exclusion** |
| --- | --- | --- | --- | --- | --- | --- |
| Asch HL, Lewis PJ, Moreland DB, Egnatchik JG, Yu YJ, Clabeaux DE, Hyland AH | Prospective multiple outcomes study of outpatient lumbar microdiscectomy: Should 75 to 80% success rates be the norm? | Journal of Neurosurgery | 2002 | 96(1) | 34-44 | - Multiple levels operated - Follow up < 1 year |
| Astrand P, Maattanen H, Vucetic N, Svensson O | Pain and orthopaedic and neurologic signs after lumbar discectomy: A 2-year follow-up | Clinical Orthopaedics and Related Research | 2000 | 379 | 154-60 | - Unclear type of discectomy, unable to contact the authors, excluded after discussion |
| Atlas SJ, Keller RB, Wu YA, Deyo RA, Singer DE | Long-term outcomes of surgical and nonsurgical management of sciatica secondary to a lumbar disc herniation: 10 year results from the Maine lumbar spine study | Spine | 2005 | 30(8) | 927-35 | - Not only microdiscectomy/open discectomy performed |
| Balaara A, Xu XF, Huang YH, Dapeng L | Predictors of the outcome of lumbar disc herniation following classical surgery: laminotomy with discectomy | Orthopade | 2017 | 46(6) | 530-537 | - Retrospective |
| Bikmullin VN, Klitsenko OA, Shulev IA | Comparative analysis of evaluating criteria for lumbar discectomy success rate | Zhurnalvoprosyneirokhirurgiiimeni N.N.Burdenko | 2012 | 76(6) | 28-35 | - Article not in English language |
| Blazhevski B, Filipche V, Cvetanovski V, Simonovska N | Predictive value of the duration of sciatica for lumbar discectomy | Prilozi | 2008 | 29(2) | 325-35 | - Unclear age, unable to contact the authors, excluded after discussion |
| DauchWA, Fass A, Brucher K | Risk factors of unsuccessful outcome after microsurgery in lumbar intervertebral disc disease | Zentralblatt fur Neurochirurgie | 1994 | 55 | 3 | - Article not in English language |
| Dorrow M, Löbner M, Stein J, Pabst A, Konnopka A, Meisel HJ, Günther L, Meixensberger J, Stengler K, König HH, Riedel-Heller SG | The course of pain intensity in patients undergoing herniated disc surgery: 5-year longitudinal observational study | PLoS One | 2016 | 11(5) | e0156647. doi: 10.1371/journal.pone.0156647. eCollection 2016 | - Not physical prognostic factors measured |
| Foidart M, Kalangu K | Prognosis of the postoperative outcome of patients with lumbar sciatica | Acta Belgica - MedicaPhysica | 1986 | 9(1) | 49-53 | - Article not in English language |
| Folman Y, Shabat S, Catz A, Gepstein R | Late results of surgery for herniated lumbar disk as related to duration of preoperative symptoms and type of herniation | Surgical Neurology | 2008 | 70(4) | 398-401 | - Retrospective |
| Goffin J | Microdiscectomy for lumbar disc herniation | Clinical Neurology & Neurosurgery | 1994 | 96(2) | 130-4 | - Retrospective - Multiple levels operated |
| Guilfoyle MR, Ganesan D, Seeley H, Laing RJ | Prospective study of outcomes in lumbar discectomy | British Journal of Neurosurgery | 2007 | 21(4) | 389-95 | - Not physical prognostic factors measured |
| Haugen AJ, Brox JI, Grøvle L, Keller A, Natvig B, Soldal D, Grotle M | Prognostic factors for non-success in patients with sciatica and disc herniation | BMC Musculoskeletal Disorders | 2012 | 13 | 183 | - Non-operative patients included |
| Hebert JJ, Fritz JM, Koppenhaver SL, Thackeray A, Kjaer P | Predictors of clinical outcome following lumbar disc surgery: the value of historical, physical examination, and muscle function variables | European Spine Journal | 2016 | 25(1) | 310-7 | - Follow up < 1year |
| Hickey OT, Burke SM, Hafeez P, Mudrakouski AL, Hayes ID, Keohane C, ButlerMA, Shorten GD | Determinants of outcome for patients undergoing lumbar discectomy: a pilot study | European Journal of Anesthesiology | 2010 | 27(8) | 696-701 | - Not only microdiscectomy/open discectomy performed - Not physical prognostic factors measured - Participants < 16 years |
| Hurme M, Alaranta H | Factors predicting the result of surgery for lumbar intervertebral disc herniation | Spine | 1987 | 12(9) | 933-8 | - Follow up < 1 year |
| Jansson KA, Nemeth G, Granath F, Jonsson B, Blomqvist P | Health-related quality of life in patients before and after surgery for a herniated lumbar disc | Journal of Bone and Joint Surgery (Br) | 2005 | 87(7) | 959-64 | - Not only microdiscectomy/open discectomy performed, unclear age |
| Johansson AC, Öhrvik J, Söderlund A | Associations among pain, disability and psychological factors and the predictive value of expectations on returning to work in patients who undergo lumbar disc surgery | European Spine Journal | 2016 | 25(1) | 296-303 | - Retrospective |
| Jönsson B | Patient-related factors predicting the outcome of decompressive surgery | Acta Orthopaedica | 1993 | 251 | 69-70 | - Not only microdiscectomy/open discectomy performed |
| Junge A, Frohlich M, Ahrens S, Hasenbring M, Sandler A, Grob D, Dvorak J | Predictors of bad and good outcome of lumbar spine surgery. A prospective clinical study with 2 years' follow up | Spine | 1996 | 21(9) | 1056-64 | - Previous surgery performed - Multiple interventions |
| Junge A, Dvorak J, Ahrens S | Predictors of bad and good outcomes of lumbar disc surgery. A prospective clinical study with recommendations for screening to avoid bad outcomes | Spine | 1995 | 20(4) | 460-8 | - Not only microdiscectomy/open discectomy performed |
| Kleinstueck FS, Fekete T, Jeszenszky D, Mannion AF, Grob D, Lattig F, Mutter U, Porchet F | The outcome of decompression surgery for lumbar herniated disc is influenced by the level of concomitant preoperative low back pain | European Spine Journal | 2011 | 20(7) | 1166-73 | - Not only microdiscectomy/open discectomy performed |
| Kohlboeck G, Greimel KV, Piotrowski WP, Leibetseder M, Krombhoh-Reindl M, Neuhofer R, Schmid A, Klinger R | Prognosis of multifactorial outcome in lumbar discectomy: A prospective longitudinal study investigating patients with disc prolapse | Clinical Journal of Pain | 2004 | 20(6) | 455-61 | - Follow up < 1 year |
| Krishnan V, Rajasekaran S, Aiyer SN, Kanna R, Shetty AP | Clinical and radiological factors related to the presence of motor deficit in lumbar disc prolapse: a prospective analysis of 70 consecutive cases with neurological deficit | European Spine Journal | 2017 | 26(10) | 2642-2649 | - Multiple levels operated |
| Lonne G, Solberg TK, Sjaavik K, Nygaard OP | Recovery of muscle strength after microdiscectomy for lumbar disc herniation: a prospective cohort study with 1-year follow-up | European Spine Journal | 2012 | 21(4) | 655-9 | - Multiple levels operated |
| Mariconda M, Galasso O, Secondulfo V et al | The functional relevance of neurological recovery after lumbar discectomy: a follow-up of more than 20 years | Journal of Bone & Joint Surgery - British Volume | 2008 | 90(5) | 622-8 | - Multiple levels operated |
| Ng LC, Sell P | Predictive value of the duration of sciatica for lumbar discectomy. A prospective cohort study | Journal of Bone & Joint Surgery - British Volume | 2004 | 86(4) | 546-9 | - Participants < 16 years |
| Rihn JA, Hilibrand AS, Radcliff K, Kurd M, Lurie J, Blood E, Albert TJ, Weinstein JN | Duration of symptoms resulting from lumbar disc herniation: effect on treatment outcomes: analysis of the Spine Patient Outcomes Research Trial (SPORT) | Journal of Bone & Joint Surgery - American Volume | 2011 | 93(20) | 1906-14 | - Retrospective |
| Schade V, Semmer N, Main CJ et al | The impact of clinical, morphological, psychosocial and work-related factors on the outcome of lumbar discectomy | Pain | 1999 | 80(1-2) | 239-49 | - Not only microdiscectomy/open discectomy performed |
| Sorensen LV, Mors O | A two-year prospective follow-up study of the outcome after surgery in patients with slipped lumbar disk operated upon for the first time | ActaNeurochirurgica | 1989 | 96(3-4) | 94-9 | - Not physical prognostic factors measured |
| Turner JA, Herron L, Weiner P | Utlity of the MMPI pain assessment index in predicting outcome after lumbar surgery | Journal of Clinical Psychology | 1986 | 42(5) | 764-9 | - Not physical prognostic factors measured - Participants < 16 years |
| Vucetic N, Astrand P, Guntner P, Svensson O, | Diagnosis and prognosis in lumbar disc herniation | Clinical Orthopaedics and Related Research | 1999 | 361 | 116-22 | - Previous surgery performed |
| Wang H, Zhang D, Ma L, Shen Y, Ding W | Factors predicting patient dissatisfaction 2 years after discectomy for lumbar disc herniation in a Chinese older cohort | Medicine (Baltimore) | 2015 | 26(10) | 2642-2649 | - Not physical prognostic factors measured |
| Wang Y, Nataraj A | Foot drop resulting from degenerative lumbar spinal diseases: clinical characteristics and prognosis | Clinical Neurology and Neurosurgery | 2014 | 117 | 33-9 | - Not a cohort study |
| Woertgen C, Holzschuh M, Rothoerl RD, Brawanski A | Does the choice of outcome scale influence prognostic factors for lumbar disc surgery? A prospective, consecutive study of 121 patients | European Spine Journal | 1997 | 6(3) | 173-80 | - Participants < 16 years |
| Woertgen C, Rothoerl RD, Holzschuh M, Breme K, Brawanski A | Are prognostic factors still what they are expected to be after long- term follow-up? | Journal of Spinal Disorders | 1998 | 11(5) | 395-9 | - Participants < 16 years |
| Woertgen C, Rothoerl RD, Breme K, Altmeppen J, Holzschuh M, Brawanski A | Variability of outcome after lumbar disc surgery | Spine | 1999 | 24(8) | 807-11 | - Participants < 16 years |
